# Universal and Versatile Magnetic Connectors for Microfluidic Devices

**DOI:** 10.3390/mi15060803

**Published:** 2024-06-19

**Authors:** Maria Alvarez-Amador, Amir Salimov, Eric Brouzes

**Affiliations:** 1 Department of Biomedical Engineering, Stony Brook University, Stony Brook, NY 11794, USA; mariajose.alv.124@gmail.com (M.A.-A.); as7580@columbia.edu (A.S.); 2 Laufer Center for Physical and Quantitative Biology, Stony Brook University, Stony Brook, NY 11794, USA; 3 Cancer Center, Stony Brook School of Medicine, Stony Brook, NY 11794, USA; 4 Institute for Engineering Driven Medicine, Stony Brook University, Stony Brook, NY 11794, USA

**Keywords:** microfluidic device connector, world-to-chip interface, thermoplastic device, glass device

## Abstract

World-to-chip interfacing remains a critical issue for microfluidic devices. Current solutions to connect tubing to rigid microfluidic chips remain expensive, laborious, or require specialized skills and precision machining. Here, we report reusable, inexpensive, and easy-to-use connectors that enable monitoring of the connection ports. Our magnetic connectors benefit from a simple one-step fabrication process and low dead volume. They sustain pressures within the high range of microfluidic applications. They represent an essential tool for rapid thermoplastic (PMMA, PC, COC) prototyping and can also be used with glass, pressure-sensitive adhesive, or thin PDMS devices.

## 1. Introduction

Thermoplastics are the materials of choice when translating research devices into commercial products. They benefit from cost-effective manufacturing techniques at high volumes, such as embossing and injection molding. Recent advances in fabrication methods allow for rapid prototyping in thermoplastics at high resolution [1,2,3,4,5,6,7,8,9,10,11,12,13]. They permit validation of designs before investing in expensive manufacturing tools. However, this toolbox does not include universal and easy-to-manufacture connectors to inject fluids into microfluidic devices.

While connecting tubing to a device is straightforward in PDMS due to its elastomeric nature, this remains challenging for rigid materials. Fast prototyping enables the testing of iterative designs and necessitates universal connectors not integrated into the device to minimize the fabrication complexity and prototyping cost.

Commercial solutions, such as the NanoPort (a plastic connector to be glued onto the device, from IDEX Health & Science), are efficient but expensive. They can be reused but involve additional manipulation, such as dissolving glue with acetone. Alternatively, integrated interference fits, or miniature sockets can sustain high pressures but require specialized tooling and precision machining [14,15]. By contrast, other investigators have used magnetic clamping to connect reconfigurable microfluidic modules [16,17,18]. In these approaches, magnetic clamping compresses an O-ring gasket to ensure sealing. Those solutions require the integration of the connectors within the device, which complicates fabrication processes. Those strategies do not accommodate the need for fast prototyping. Notably, a magnetic connector can be made by gluing a capillary tubing inside a magnet ring and an O-ring gasket on the opposite face to create an effortless connection [19]. However, the multi-step fabrication includes the usage of glue, limits the tubing magnet ring combination, generates opaque connectors with high dead volume, and does not provide simple alignment with fluidic ports.

This report presents a simple magnetic microfluidic connector that can be easily fabricated in a single step in any laboratory. Our solution is a magnetic ring overfilled with PDMS. The PDMS elasticity seals the tubing, and the PDMS-device interface is sealed through magnetic clamping. Our magnetic connectors do not require an O-ring gasket or glue that can wick in and block microfluidic channels. Our solution is cost-effective, flexible, and easy to use. We demonstrate that our magnetic connectors are leak-free at injection pressures above 45 psi across various materials, including polymethyl methacrylate (PMMA), polycarbonate (PC), cyclic olefin copolymer (COC), pressure-sensitive adhesive (PSA), glass, and thin polydimethylsiloxane (PDMS), highlighting their utility and versatility. We also establish that their sealing pressure directly correlates with the clamping pressure. Finally, we prove the robustness of our magnetic connectors by generating microfluidic droplets for several hours with an applied backpressure that varies across a wide range of pressures (0–24 psi).

Our design delivers leak-free connectors that are easy to fabricate and low-cost. The low dead volume connectors can easily align with microfluidic ports and enable their monitoring. Our magnetic connectors fill a gap for rapid prototyping in thermoplastics (PMMA, PC, COC), glass, and thin PDMS.

## 2. Materials and Methods

### 2.1. Magnetic Connectors

Magnetic rings (Table 1) were purchased from K&J Magnetics (Pipersville, PA, USA). PDMS (Sylgard 184 Silicone Elastomer Kit, Dow Corning, Midland, MI, USA) was mixed at a 10:1 (polymer: crosslinker) weight ratio and poured into the rings. The assembly was cured at 65 °C in an oven for 2 h. The PDMS insert was punched with a biopsy tool (Syneo, West Palm Beach, FL, USA) mounted on a mini drill press (TBM 220, Proxxon, Hickory, NC, USA) to use its lever motion. PEEK tubing (0.254 mm inner diameter (ID), 0.787 mm outer diameter (OD), Zeus, Orangeburg, SC, USA) was inserted through the hole, and its extremity was cut clean but not flush to allow easy alignment with the port.

### 2.2. Materials Tested

The materials tested consisted of 1 mm thick flat pieces drilled to create a series of 1 mm ports. One side of the ports was sealed with pressure-sensitive adhesive (PSA) tape. The materials used included cyclic olefin copolymer (COC-Topas 5013L-10, Tg = 134 °C, Knightsbridge Plastics Incorporated, Fremont, CA, USA), polycarbonate (PC, 8574K19, McMaster Carr Supply Company, Chicago, IL, USA), and polymethyl methacrylate (PMMA, ME30-SH-000117, Goodfellow Corporation, Pittsburgh, PA, USA). Glass (Premium Plain Micro Slides, 48300-026, from VWR International, Pittsburgh, PA, USA), PSA tape film (ThermaSeal RTS, Thomas Scientific, purchased from Merck KGaA, Darmstadt, Germany), and Polydimethylsiloxane (PDMS, 10:1 polymer:crosslinker wt., Sylgard 184, Dow Chemicals, Midland, MI, USA) were tested without injection ports. The thin PDMS layer was cast over a 22 mm × 40 mm glass coverslip (Fisherbrand, Thermo Fisher Scientific, Waltham, MA, USA), which provided mechanical stability. The thickness of the PDMS sample was 1 mm, including the coverslip. By conducting a series of experiments with COC without ports, we ensured that the presence of ports does not influence the value of the sealing pressure.

### 2.3. Droplet Generator Microfabrication

We fabricated cyclic olefin copolymer (COC) devices using 5:1 wt. PDMS embossing dies obtained by soft lithography [20], following a published protocol [2,21]. A 3″ silicon wafer (P(100) 0–100 ohm cm SSP 406–480 μm test grade silicon wafer, UniversityWafer Inc., Boston, MA, USA) is first patterned with the negative photoresist SU-8 (Kayaku Advanced Materials, Westborough, MA, USA) and silanized overnight by vapor deposition with a fluorinated silane compound (1H,1H,2H,2H-Perfluorooctyltrichlorosilane, Gelest, Morrisville, PA, USA). After molding and curing, the 5:1 wt. PDMS embossing die is silanized overnight by vapor deposition with the fluorinated silane compound after activation with a handheld corona generator (BD-20AC, Electro-Technic Products, Chicago, IL, USA). A 50 mm × 75 mm glass slide is stacked with the 1 mm thick COC thermoplastic plate, the 5:1 PDMS die, a glass slide, and a layer of density foam (1059N372, medium hardness, 16 psi to compress 25%, Mc Master-Carr, Elmhurst, IL, USA). The sandwich is clamped between two aluminum plates with office binders (Figure S1). The assembly is incubated at 150 °C in a conventional oven overnight. The assembly is allowed to cool to room temperature before drilling 1 mm diameter ports. The device is then manually sealed with a PSA tape (ThermalSeal RTS, Thomas Scientific, Swedesboro, NJ, USA).

### 2.4. Sealing Pressure Measurement

Solutions were injected into microfluidic devices via PEEK tubing using a pressure controller (MPV1 models, Proportionair, McCordsville, IN, USA) operated with a custom-built software developed under LabVIEW 2014 (version 14.0.1f11, National Instruments, Austin, TX, USA) via an Arduino UNO interface (Adafruit Industries, New York, NY, USA). The pressure controller could deliver up to 60 psi. Microfluidic chips were placed on an inverted microscope (Diaphot-TMD, Nikon, Tokyo, Japan) and observed using a 10× (Ph1 10/0.30 DL 160/0.17, Nikon) or a 20× objective lens (Ph2 20/0.40 ELWD 160/1.2, Nikon) under bright-field illumination. Images were recorded with a CCD camera (XCD-V60, Sony, Tokyo, Japan) using custom-built software developed under LabVIEW.

The tubing was primed with a solution of blue food dye diluted in water and filtered. For each test, pressure was gradually increased by 1 psi every 100 recorded frames or 30 s until a leak was detected.

### 2.5. Sealing Pressure vs. Magnetic Pressure

The magnetic force was modulated by inserting COC shims to vary the distance between the magnetic rings. This distance, which includes the dome height, was measured with a caliper. The pull-up force as a function of the distance was obtained using a magnet calculator (K&J magnetics, https://www.kjmagnetics.com/calculator.asp, accessed on 17 June 2024).

We collected at least 4 measurements for each magnetic force. We reported the measurements using a scatter plot to reflect the variation in the dome height between different magnetic connectors.

### 2.6. Droplet Generation and Long-Term Stability

Microfluidic droplets were generated with a 100 μm wide and 45 μm deep COC/PSA droplet generator. Fluids were injected with syringe pumps (Nemesys, CETONI, Korbussen, Germany) using gastight 1 mL syringes (Hamilton, Reno, NV, USA) at 150 μL/h for the oil phase (2% wt. PFPE-Krytox surfactant [22,23] in FC 3283 (3M, St. Paul, MN, USA)) and 100 μL/h for the water phase. The backpressure was adjusted every 15 min using a pressure controller. Droplets were generated over 3 h, and 500 images were collected every 15 min using a 4× objective (E Plan 4/0.1, Nikon) on the inverted microscope. Images were processed with the FIJI distribution of ImageJ to extract the evolution of the droplet size over time [24]. We report the number of droplets detected and analyzed in each series of 500 images as a proxy for droplet throughput.

## 3. Results

### 3.1. Simple Design and Fabrication

**Figure 1:**
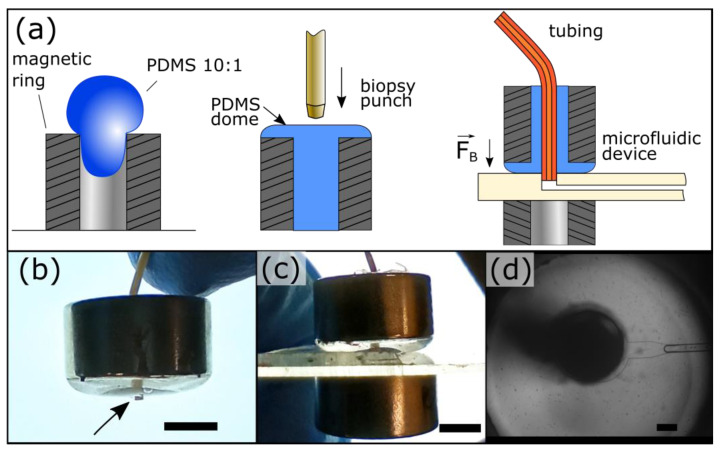
Microfluidic magnetic connectors. (**a**) The fabrication of the magnetic connectors is simple and inexpensive and can be performed in any laboratory. A magnetic ring is filled with PDMS, cured, and then punched. (**b**) The magnetic connector self-guides into ports by using a short tubing overhang (arrow). (**c**) The bottom ring magnet self-mates with the top for easy clamping. (**d**) The port can be easily observed through the PDMS. The port is shown here with a ¼″–⅛″ outer–inner diameters magnetic connector. The scale bar represents 1 mm in panels (**b**) and (**c**) and 200 μm in panel (**d**).

Our magnetic connectors are fabricated by filling rare Earth neodymium magnetic rings with PDMS. They harness the PDMS elasticity to provide sealing at two interfaces: (1) between the PDMS and the tubing and (2) between the PDMS and the device material (Figure 1a). The latter is secured by magnetic clamping using a second magnetic ring beneath the device. Using a short tubing overhang, the magnetic connector self-guides into the port (Figure 1b,c). The overhang is fitted within the port before applying the bottom magnet, which self-mates with the top magnetic ring. This approach is efficient straightforward, and does not require experience. The PDMS clarity allows users to observe ports during operation (Figure 1d), which is critical to monitor device start-up and the presence of contaminants. A complete 3/8″ magnetic connector costs less than $3 ($1.28 per magnetic ring and $0.3 for PDMS), 10 times less than commercial solutions. In addition, their fabrication requires minimal equipment and hands-on time. These magnetic connectors can be reused by changing the tubing to avoid cross-contamination or recast if the PDMS is worn out.

### 3.2. Robust Sealing Performances

We measured the sealing pressure at which the magnetic connectors started leaking to establish their working pressures. We used simple ports without outlets and increased the injection pressure until leakage was observed. For that purpose, we tested a wide range of materials, including glass, PMMA, PC, COC, PSA, and thin PDMS. These materials encompass the most used materials in microfluidics, with surface properties ranging from hydrophilic to hydrophobic. PSA can also be applied to many materials and constitute an efficient universal pad for our magnetic connectors. We also tested the efficacy of two ring magnets of different sizes and pull-up forces in maintaining a seal. The thickness of all the materials tested was 1 mm. A solution of dyed water was injected into the channel at increasing injection pressures until leakage. The experimental setup was observed under a microscope during injection. Leaks can occur at any of the three interfaces of the system: (1) tubing and PDMS, (2) PDMS and material, and (3) material and PSA. Leaks were only observed at the PDMS–material interfaces.

**Figure 2:**
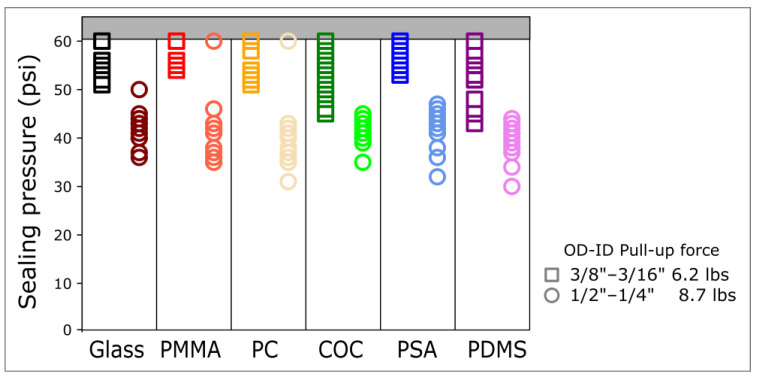
Sealing pressure of magnetic connectors across a wide range of materials. The sealing pressure was measured for glass, polymethyl methacrylate (PMMA), polycarbonate (PC), cyclic olefin copolymer (COC), pressure-sensitive adhesive (PSA), and polydimethylsiloxane (PDMS) using two ring magnets of different sizes and pull-up forces. The thickness of all the materials tested was 1 mm. The highest injection pressure was limited to 60 psi. Per material, data were generated with at least 3 different connectors and data points were repeated 3 times for each connector.

The 8.73 lbs ½″ outer diameter (OD) magnetic connectors allow for sealing pressures of up to 45 psi. In comparison, the 6.2 lbs 3/8″ OD magnetic connectors allow for sealing pressures above 50 psi across all the materials tested (Figure 2). These sealing pressures are comparable to burst pressures of PSA to COC, PDMS to glass, or PDMS to PDMS, which are 72 psi [11], 74 psi [25], and 58 psi [26], respectively.

**Figure 3:**
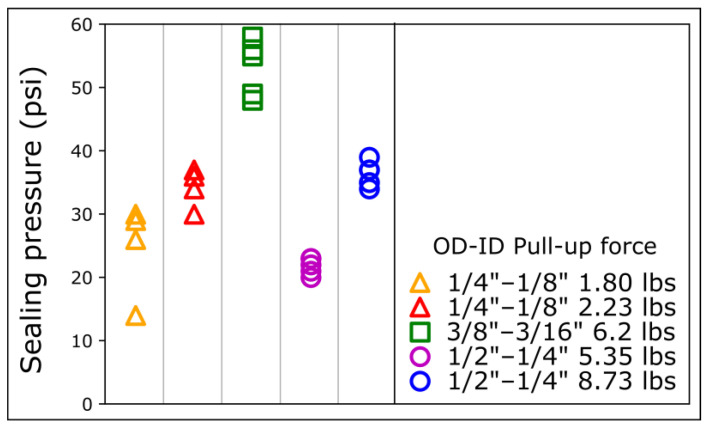
Sealing pressures for different magnetic connectors on COC. The sealing pressure is measured for magnetic connectors of different sizes and pull-up forces. OD: outer diameter; ID: inner diameter. For each magnet ring type, data points were repeated 4 times with different magnetic connectors.

Interestingly, despite a lower pull-up force, the 6.2 lbs 3/8″ OD connectors provided a higher sealing pressure than the 8.73 lbs ½″ OD connectors. To further elucidate the relationship between sealing pressure, pull-up force, and diameter, we measured the sealing pressure of magnets of different sizes and pull-up forces on COC (Figure 3). Despite a much lower pull-up force, the 2.23 lbs ¼″ OD and the 1.8 lbs ¼″ OD connectors provided a sealing pressure comparable to the 8.73 lbs ½″ OD and 5.35 lbs ½″ OD, respectively (Figure 3).

To explain these intriguing results, we hypothesized that the sealing pressure was a function of the magnetic clamping pressure, defined as the magnetic force divided by the magnet surface area. To test this hypothesis, we modulated the magnetic force by inserting COC shims between the magnetic rings. We reported the sealing pressure as a function of the clamping pressure. We measured the sealing pressure for each magnetic ring on COC for five different distances.

**Figure 4:**
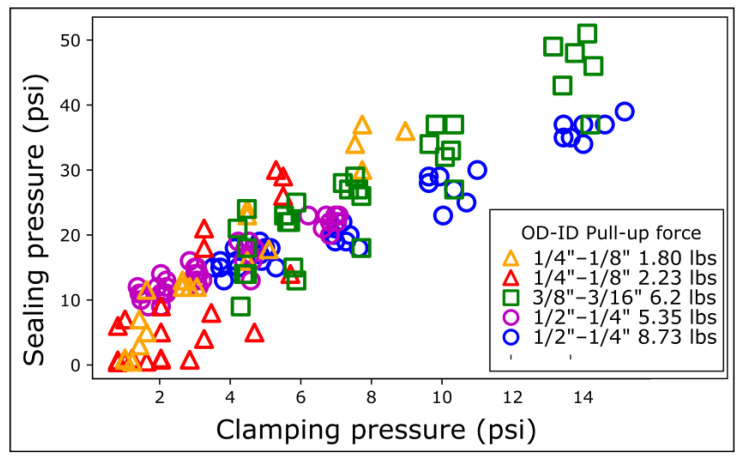
Sealing pressure as a function of magnetic clamp pressure on COC. The clamping pressure is defined as the clamping force divided by the outer surface area of the magnet. The clamping force is modulated by varying the inter-magnet distance using shims. Each series of distances was repeated 5 times with different magnetic connectors.

The sealing pressure increases linearly with the clamping pressure. The data for the different OD magnetic connectors align roughly on the same master curve but still have some significant spread (Figure 4). Data from magnetic rings with the same OD align better with each other, underlining the need to consider the exact contact surface area. The narrower ¼″ OD magnetic connectors exhibit variability in sealing pressure at low clamping pressures (0–4 psi, Figure 4). The spread of the data for the narrowest magnetic connectors is attributed to the lower mechanical stability of the contact at low magnetic clamping pressure. More precisely, the PDMS dome at the top of the magnetic ring is rounder for the narrowest connectors, making them sensitive to the torque generated by the tubing (Figure S2).

**Figure 5:**
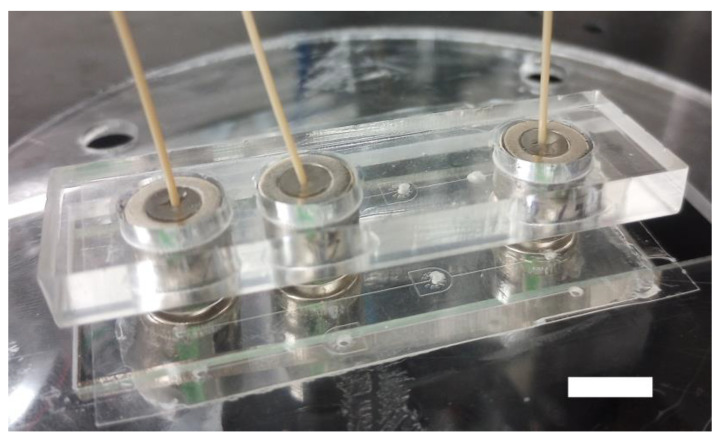
Stabilizing jig. 3/8″ OD magnetic connectors are stabilized with 5.5 mm thick laser-cut acrylic jigs. The scale bar represents 10 mm.

**Figure 6:**
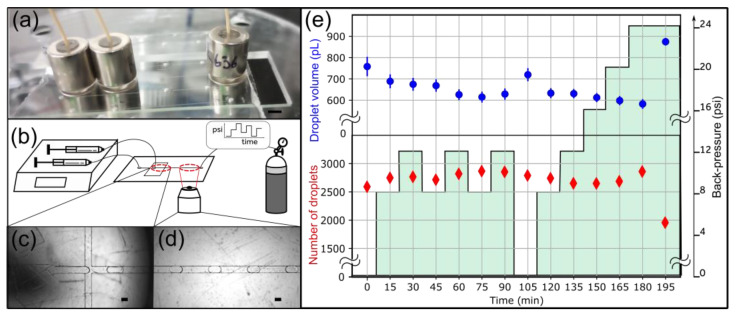
Droplet generation experiment to evaluate the long-term stability of 6.2 lbs 3/8″ OD magnetic connectors. (**a**) A side view of the device shows the arrangement of magnetic connectors. The scale bar represents 5 mm. (**b)** Experimental setup. Fluids are injected with syringe pumps. An adjustable backpressure is applied at the outlet using a pressure controller. (**c**) Image of the nozzle. (**d**) Typical image of droplets in the collection channel. Scale bars represent 100 μm in panels c and d. (**e**) The droplet volume (blue) and the number of droplets detected and analyzed (red) are plotted over time. The evolution of the backpressure is represented in green.

Altogether, these data demonstrate that the range of sealing pressures for the magnetic connectors covers the working range of typical microfluidic applications very well. They also allow us to predict the expected sealing pressure for those magnet sizes. From a practical point of view, using several magnetic connectors imposes dimensional constraints on the microfluidic design due to their footprint. We can use smaller magnet rings, such as the ¼″ OD, and reach the same sealing pressures, which permits a higher density of ports on a device. Additionally, rare Earth neodymium magnets interact strongly with each other. To prevent uncontrolled magnet aggregation, magnetic connectors for adjacent ports should be placed into a 3D-printed jig (Figure 5) or in direct contact (Figure 6a). Notably, jigs can also prevent the smaller magnetic connectors from rocking under the torque that the tubing imposes at a lower magnetic clamp pressure. Jigs are necessary if 3/8″ magnetic connectors are within 10 mm or less due to long-range magnetic interactions (Figure S3). From a practical point of view, the 3/8″ magnetic connectors are easier to manipulate due to their combination of pull-up force and height, which allow a better grasp. The sealing performances of the magnetic connectors are comparable to typical bonding values, which means that the magnetic connectors will not be the weakest element for high-pressure applications. Future improvements include investigating the best magnetic ring pairs for an optimal aspect ratio and sealing pressure to improve handling, especially for multiple ports applications.

### 3.3. Long-Term Stability of the Magnetic Connectors

Next, we sought to characterize the performance of magnetic connectors at longer timescales. We tested their practical utility for droplet generation, which is sensitive to flow perturbation. A small leak in the system would translate into detectable droplet size and throughput fluctuations [11,27]. Using two syringe pumps, we injected water and a fluorinated oil solution into a COC/PSA droplet generator. We imposed varying backpressure to challenge the magnetic connectors at the inlets (Figure 6b). We generated droplets for over 3 h and recorded 500 images every 15 min (Figure 6c,d). The backpressure cycled between 8 and 12 psi before returning to 0 psi and increasing stepwise up to 24 psi (Figure 6e). We used the 6.2 lbs 3/8″ OD magnetic connectors in these experiments.

The droplet volume remained stable during the experiment (Figure 6e). The standard deviation at each data point was around 4.0%, and the standard deviation of the average droplet volume across all time points was around 6.1% with all the data points and 4.5% without the 0 psi data. This variation is expected due to the fluctuations in flow rate, which ranges within a few percent for our syringe pumps. Thus, the variation in droplet volume can be attributed to the variability of droplet generation and not to a leak. The data at 0 psi repeated within the standard deviation after cycling three times the backpressure between 8 and 12 psi (Figure 6e, data points t = 0 min and t = 105 min). The 0 psi data points deviate from the other data points, which may be explained by mechanical deformation at higher backpressures. The data point at 195 min deviates significantly from the rest due to a leak at the PSA-COC interface. This data point validates the ability of our setup to detect leaks. The number of droplets analyzed at each time point, used as a proxy for throughput, was also stable throughout the 180 min experiment. We conclude that the system did not leak at the magnetic connectors for 3 h, demonstrating their long-term stability.

The sealing pressure depends on the wettability of the device material by the injected solutions. For instance, fluorinated oil has a lower surface energy than water, and its sealing pressure is expected to be lower. Despite this limitation, droplet generation was stable for 3 h with variable backpressures in the high-pressure range for typical microfluidic applications.

**Figure 7:**
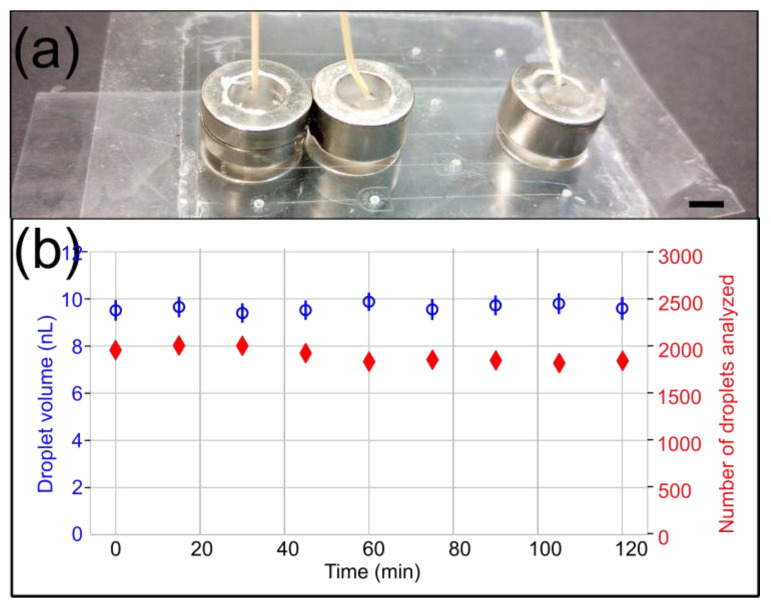
Droplet generation experiment with a 10 psi backpressure to evaluate the long-term stability of 8.73 lbs ½″ OD microfluidic magnetic connectors. (**a**) A side view of the device shows the arrangement of magnetic connectors. The scale bar represents 5 mm. (**b**) The droplet volume (blue) and the number of droplets detected and analyzed (red) are plotted over time. The data demonstrate the stability and absence of leakage over 2 h.

The PSA-COC interface delaminated at a lower pressure than reported in the literature [11]; however, we already noticed that fluorinated oil weakens the bond between PSA and COC [21]. Finally, we also stably generated microfluidic droplets with a 10 psi backpressure for 2 h using the 8.73 lbs ½″ OD magnetic connectors (Figure 7).

## 4. Conclusions

Connecting tubing to microfluidic chips remains challenging for rigid devices. We report simple, reusable connectors combining PDMS sealing with magnetic clamping. They are straightforward to interface with microfluidic devices and can be easily fabricated in a single step with minimal skills. The magnetic connectors are quick to connect, easy to align, and offer low dead volume. Their sealing exhibited excellent and predictable performance. The magnetic connectors maintain sealing over the pressure range typical of most microfluidic applications for long periods. They are universal, inexpensive, reusable, and can be used with various device materials, including PMMA, PC, COC, glass, PSA, and thin PDMS.

## 5. Note

For further proof of the utility of our magnetic connectors, we used them to inject an aqueous solution at an injection pressure ranging from 3 to 24 psi into a COC-PSA partitioning platform [27].

## Figures and Tables

**Table 1:** Magnetic rings tested in the study.

Magnet Reference Number	Outer Diameter (OD) (Inch)	Inner Diameter (ID) (Inch)	Thickness (Inch)	Pull-Up Force ^§^ (lbs)
R422	1/4	1/8	1/8	1.8
R422-N52	1/4	1/8	1/8	2.23
R636	3/8	3/16	3/8	6.2
R842	1/2	1/4	1/8	5.35
R844	1/2	1/4	1/4	8.73

^§^: Pull-up force between a pair of identical magnets.

## Data Availability

Data can be requested from the corresponding author.

## Supplementary Materials

The following supporting information can be downloaded at: https://www.mdpi.com/article/10.3390/mi15060803/s1, Figure S1: COC imprinting, Figure S2: Shape of PDMS domes, Figure S3: Inter-magnetic connectors stability.
