# Supplementary material for: Universal and Versatile Magnetic Connectors for Microfluidic Devices
Source: Micromachines (Basel). 2024 Jun 19;15(6):803. doi: 10.3390/mi15060803 (PMC11205433; doi:10.3390/mi15060803)
Supplement: Supplementary file 1 [file micromachines-15-00803-s001.zip › micromachines-3049316-supplementary.pdf]

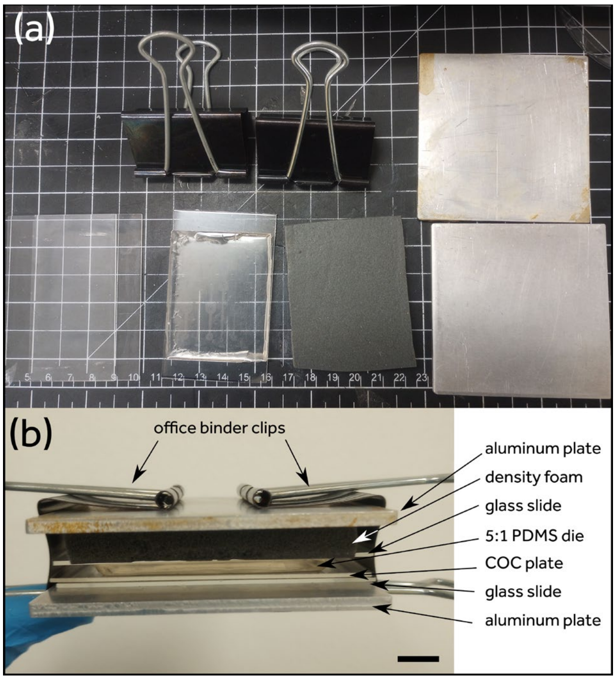

Figure S1: COC imprinting. (a) Parts needed to imprint a COC plate with a 5:1 PDMS die. (b) Sandwich assembly to be incubated overnight in a conventional oven at 150 C. The scale bar represents 10 mm.

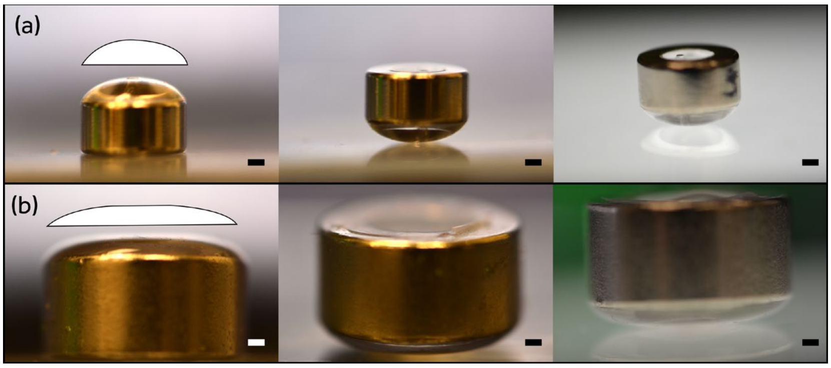

Figure S2. Shape of PDMS domes. (a) ¼″ OD magnetic connectors exhibit a spherical cup shape, which creates a small contact area. (b) ½″ OD magnetic connectors exhibit a flatter dome, which increases their stability. The contours of the domes are represented in a white shape in the first panels. Scale bars represent 1 mm.

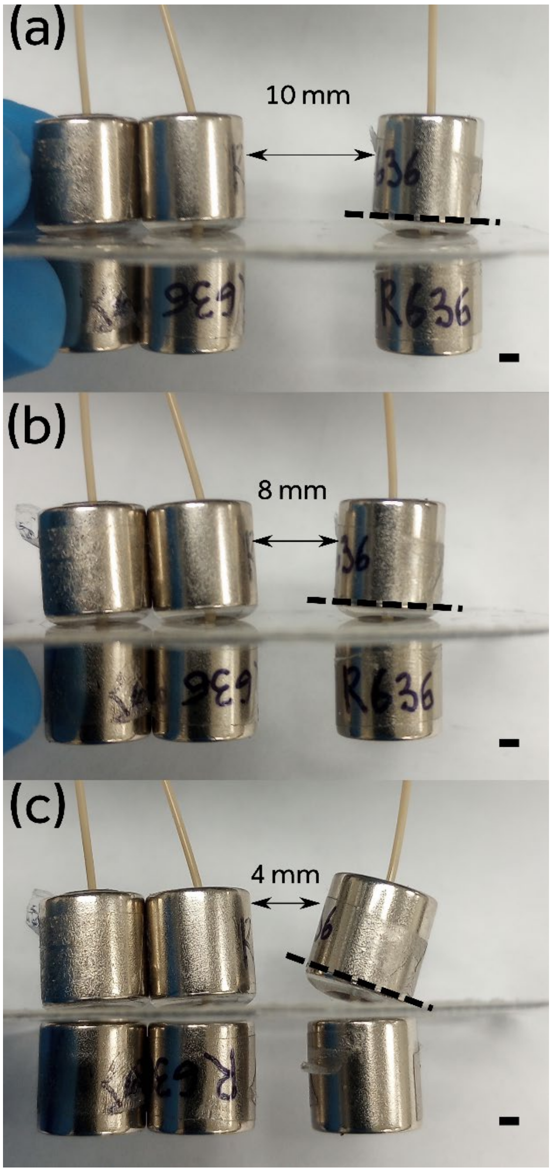

Figure S3: Inter-magnetic connectors stability. (a) Magnetic connectors do not influence each other enough to tilt the base of the magnetic connector when separated by 10 mm or more. (b) The magnetic of neighboring magnetic connectors starts around 8 mm. (c) Strong effect of a neighboring magnetic connector at 4 mm. At this distance, the magnetic connector is tilted and cannot maintain sealing. The scale bars represent 1 mm.
